# P-908. Treatment Strategies and Outcomes for Carbapenem-Resistant Acinetobacter baumannii and Stenotrophomonas maltophilia Infections Before and After Institutional Treatment Guidance Implementation

**DOI:** 10.1093/ofid/ofaf695.1114

**Published:** 2026-01-11

**Authors:** Ryan Vathy, Christo Cimino, Ben Ereshefsky, Romney Humphries

**Affiliations:** Vanderbilt University Medical Center, Nashville, Tennessee; Vanderbilt University Medical Center, Nashville, Tennessee; Vanderbilt University Medical Center, Nashville, Tennessee; Vanderbilt University Medical Center, Nashville, Tennessee

## Abstract

**Background:**

The Infectious Diseases Society of America guidance document on the treatment of multidrug-resistant (MDR) infections was first published in December 2021. This document addressed the treatment of carbapenem-resistant Acinetobacter baumannii (CRAB) and Stenotrophomonas maltophlia amongst other organisms. At our institution, a treatment algorithm was shared with ID providers on June 1st, 2022, consistent with IDSA’s recommendations. This treatment algorithm reflected the most up to date dosing and treatment options for CRAB and *S. maltophilia.* The objective of this study was to evaluate if this treatment guidance document impacted treatment decisions and clinical outcomes for infections caused by CRAB or S. maltophilia at our institution.

**Methods:**

This project was a single-center, retrospective, quasi-experimental, pre- and post-intervention study of patients who were admitted from Vanderbilt University Hospital from November 1st, 2018 to June 1st, 2024, who had growth of CRAB or S. maltophilia on any culture and received definitive treatment. The primary outcome was compliance with the institutional guidance document. Secondary outcomes included clinical efficacy, hospital readmission within 90 days, all-cause 30-day mortality, 90-day microbiologic recurrence, and antibiotic associated adverse events. Continuous variables were compared using a Mann-Whitney test and categorial variables were compared using a Pearson’s Chi-Square test.

**Results:**

Twenty-two patients with CRAB (14 pre and 8 post-intervention) and 150 with S. maltophilia (75 pre and 75 post-intervention) were evaluated. For CRAB, 0 (0%) patients in the pre-intervention group and 5 (62.5%) in the post-intervention group met the primary outcome (p < 0.001). For S. maltophilia, 21 (28%) patients in the pre-intervention group compared to 38 (50.7%) in the post-intervention group met the primary outcome (p < 0.004). Secondary outcomes did not differ significantly.

**Conclusion:**

These findings highlight that implementing local treatment guidelines can lead to increased adherence to guideline directed antimicrobial therapy.

**Disclosures:**

All Authors: No reported disclosures

